# Inter-Cellular Transport of Ran GTPase

**DOI:** 10.1371/journal.pone.0125506

**Published:** 2015-04-20

**Authors:** Deepak Khuperkar, Mary Helen, Indrasen Magre, Jomon Joseph

**Affiliations:** National Centre for Cell Science, Ganeshkhind, Pune, India; Indian Institute of Science, Bangalore, INDIA

## Abstract

Ran, a member of the Ras-GTPase superfamily, has a well-established role in regulating the transport of macromolecules across the nuclear envelope (NE). Ran has also been implicated in mitosis, cell cycle progression, and NE formation. Over-expression of Ran is associated with various cancers, although the molecular mechanism underlying this phenomenon is unclear. Serendipitously, we found that Ran possesses the ability to move from cell-to-cell when transiently expressed in mammalian cells. Moreover, we show that the inter-cellular transport of Ran is GTP-dependent. Importantly, Ran displays a similar distribution pattern in the recipient cells as that in the donor cell and co-localizes with the Ran binding protein Nup358 (also called RanBP2). Interestingly, leptomycin B, an inhibitor of CRM1-mediated export, or siRNA mediated depletion of CRM1, significantly impaired the inter-cellular transport of Ran, suggesting a function for CRM1 in this process. These novel findings indicate a possible role for Ran beyond nucleo-cytoplasmic transport, with potential implications in inter-cellular communication and cancers.

## Introduction

The well-structured nucleus helps the eukaryotic cells to achieve a fine-tuned regulation of gene expression, but demands the cell to have mechanisms in place to coordinate the transport of macromolecules across the nuclear membrane for effective nuclear-cytoplasmic communication and cell homeostasis. One of the major pathways regulating nuclear import and export involves the GTPase Ran [1–4]. The asymmetric localization of Ran’s regulators—the guanine nucleotide exchange factor RCC1 in the nucleus [5] and the GTPase activating protein RanGAP1 in the cytoplasm [6,7]—primarily generates a Ran GTP gradient across the NE [8], which dictates the directionality of nuclear transport [9]. One of the well-studied transport processes is mediated through RanGTP-binding transport receptors called importins and exportins [10]. The import complex, consisting of the cargo protein that possesses the nuclear localization signal (NLS) and the import receptors (importin α/β), is assembled in the cytoplasm, and is transported through the nuclear pore complex (NPC) into the nucleus. Binding of RanGTP to importin β displaces and releases the cargo inside the nucleus. Conversely, the export complex is formed in the nucleus by the trimolecular association between the cargo that possesses the nuclear export signal (NES), exportin1 (also called CRM1) and RanGTP, which upon reaching the cytoplasm through the NPC, is disassembled as a consequence of RanGAP1-mediated hydrolysis of GTP bound to Ran [1,3]. Some transport receptors also help in localizing different RNA species/RNA protein complexes into the nucleus or to the cytoplasm. For example, Snurportin1 mediates nuclear import of spliceosomal UsnRNPs in an importin β-dependent manner [11] and exportin-5 is an adapter for miRNA export from the nucleus to cytoplasm [12–14]. Exportin-1 aids in the export of several UsnRNAs, a subset of mRNAs, and assembled ribosome subunits from the nucleus [15–17]. Similarly, Exportin-t is an adapter used in the export of tRNAs from the nucleus to cytoplasm in a RanGTP-dependent manner [18,19]. Apart from the well-defined function in nuclear transport, Ran GTPase also plays critical roles in mitosis, cell cycle progression and NE reformation, through a mechanism similar to that employed in nucleo-cytoplasmic transport [20–23].

In addition to the intra-cellular signalling, multi-cellular organisms also evolved robust inter-cellular communication system to coordinate different processes during growth, development and adult homeostasis. One of the well appreciated forms of such communication is initiated by specific binding of a ligand secreted by one cell to the transmembrane receptor present on the recipient cell, and subsequent relay of signalling through defined protein-protein and protein-nucleic acid interactions [24]. Recent studies have identified other modes of cell-cell communication to include distribution of molecules between cells through tunnelling nanotubes (TNTs) [25,26] and microvesicles (exosomes and shedding vesicles) [27–29]. TNTs are inter-cellular actin-rich connections implicated in the inter-cellular transfer of molecules and organelles in cultured cells. However, the evidence for existence of TNTs in tissues is lacking [25]. In addition to TNTs, inter-cellular macromolecule distribution also occurs through secreted vesicles generally termed as microvesicles. Whereas exosomes are vesicles derived from multivesicular bodies, the shedding vesicles are generated by the direct budding from the plasma membrane. These microvesicles are shown to contain a plethora of proteins, mRNAs and miRNAs [29–31]. Interestingly, TNTs and microvesicles are shown to function in immune cell signalling and cancer progression [28,29,32–34]. Additionally, a class of proteins, including homeoproteins, is shown to exhibit inter-cellular movement through a mechanism involving non-conventional secretion and internalization [35,36].

Here we report that Ran GTPase possesses the ability to get transferred between cultured mammalian cells. The distribution is GTP-dependent and requires the export receptor CRM1.

## Methods

### Cell Culture, Reagents and Treatments

HeLa S3, NIH3T3, COS-7 and HEK293T cells were grown in Dulbecco’s Modified Eagle’s Medium (DMEM) with 10% Fetal Bovine Serum (FBS) and antibiotics at 37°C in a humidified atmosphere with 5% CO_2_. tsBN2 cells (a kind gift from Mary Dasso, NIH, USA) were regularly grown in DMEM and 10% FBS at 32.5°C (permissive temperature) with 5% CO_2_. For experiments with depleted RCC1, tsBN2 cells were shifted to 39.5°C (non-permissive temperature) for indicated time points.

Rabbit polyclonal antibodies against Nup358 and GFP have been described earlier [37,38]. Rat anti-HA (Roche, 1:100) or mouse anti-HA (Covance, 1:3000) was used for immunostaining. Secondary antibodies used for immunofluorescence were goat anti-rat 350, goat or donkey anti-rabbit 488, donkey anti-mouse 594 (Invitrogen, 1:1000). Hoechst-33342 dye (Sigma-Aldrich) was used to stain the DNA. Western blotting was performed with mouse anti-GFP (Santa Cruz Biotechnology, sc-9996, 1:3,000), mouse anti-Ran (BD biosciences, 610340, 1:10,000), goat anti-RCC1 (Santa Cruz Biotechnology, sc-1161, 1: 3000), mouse anti-CRM1 (BD biosciences, 611832, 1:1500) and mouse anti-α-tubulin (Sigma-Aldrich, T5168, 1:5,000) antibodies.

Leptomycin B (LMB) was purchased from Sigma-Aldrich. After transfection, cells were treated with 5 ng/ml of LMB for indicated time points.

### DNA constructs

The open reading frames (ORFs) of human Ran mutants Q69L and T24N were amplified by PCR from pET30-Ran-Q69L and pcDNAI-Ran-T24N (kind gifts from Mary Dasso, NIH, USA), respectively, and subcloned into EcoRI site of pEGFP-C1 (Clontech). pEGFP-Ran-G19V and pEGFP-Ran-wild type (WT) were generated by subcloning the respective ORFs from pKH3-Ran constructs (generously provided by Ian Macara, Univ. Virginia, USA). The inserts released by BamHI-EcoRI digestion were subsequently cloned into BglII-EcoRI sites of pEGFP-C1 (Clontech). To generate 2xGFP, 2xGFP-Ran-Q69L and 2xGFP-Ran-T24N, GFP ORF was released from pEGFP-C1 by NheI (klenow-endfilled)-HindIII digestion and cloned at BsrGI (klenow-endfilled)-HindIII sites of pEGFP-C1, pEGFP-Ran-Q69L and pEGFP-Ran-T24N, respectively. BamHI-EcoRI fragment from pKH3-Ran wild type was subcloned into BglII-EcoRI sites of 2xGFP vector to generate 2xGFP-Ran-WT.

pcDNA3.1-mCherry-α-tubulin (a kind gift from Frederic Saudou, Institut Curie, France) and HA-GAPDH (generously provided by Akira Sawa, Johns Hopkins University School of Medicine, USA) were used in transient transfections as transfection markers. pEGFP-Cdc42 mutants were provided by Francisco Sanchez-Madrid (Universidad Autonoma de Madrid, Spain).

### Transfections

HeLa cells were grown on glass coverslips in a 24-well plate for 12 h and were transfected with indicated constructs using polyethylene imine (Polysciences, Inc.) or Lipofectamine 2000 as per manufacturer’s instructions.

For co-culturing experiments, initially, 1 x 10^**5**^ HeLa cells (donor) and 1 x 10^**5**^ NIH3T3 cells (recipient) were plated separately in each well of a 24-well plate. After 12 hours, transfection was performed in HeLa cells and 9 h later, both HeLa and NIH3T3 cells were trypsinized, mixed in the ratio of 1:5 and plated on glass coverslips. Eighteen hours later, the coverslips were analysed by fluorescence microscopy.

For transient transfections, 1 x 10^**5**^ HeLa cells were plated on coverslip in each well of 24-well plates. After 12 h, cells were transfected with indicated DNA constructs with or without either of the transfection markers, mCherry-α-tubulin or HA-GAPDH as indicated. Nine hours post-transfection, coverslips were analysed by fluorescence microscopy.

For nucleofection, 3 x 10^**5**^ HeLa cells were trypsinized and transfected with indicated DNA constructs (400 ng) using Amaxa nucleofector (Lonza) according to manufacturer’s instructions (High Viability program). The transfected cells were plated on coverslips. Twenty four hours post-transfection, coverslips were analysed by fluorescence microscopy.

### Fluorescence confocal microscopy

In all experiments, except wherever indicated, cells were fixed using methanol and processed for immunofluorescence microscopy as described earlier [39]. Alternatively, cells were fixed with 2% PFA in phosphate-buffered saline (PBS) for 20 min, permeabilized with 0.5% triton X-100 in PBS for 10 min and incubated with 10% normal horse serum before proceeding with immunostaining. Coverslips were mounted in VECTASHIELD mounting medium (Vector Laboratories) for confocal imaging. The images were obtained using a laser-scanning confocal microscope (TCS SP5; Leica) with a Plan Apochromat 63.0x objective (1.40 NA, oil) with similar settings and were processed using Photoshop using similar parameters / settings (CS2; Adobe).

### Western Blotting

HEK293T cells expressing the indicated proteins were lysed in RIPA buffer [50 mM Tris-HCl (pH 8.0), 150 mM NaCl, 1% NP-40, 0.5% sodium deoxycholate, 0.1% SDS, supplemented with protease inhibitor cocktail (Roche), 10 mM NaF, 2 mM PMSF]. Protein estimation was carried out and equal amount of proteins were subjected to SDS-PAGE. The proteins were transferred to PVDF membrane (Millipore), and western blotting was performed using indicated antibodies.

### Quantitation of Ran transfer and statistical analysis

In all the experiments, except wherever indicated, the cells were transfected, fixed, stained, mounted and directly visualized under Zeiss Axiovert 200M microscope with a 63x objective (Plan Apochromat, 1.4 NA, oil) and the cells that showed detectable fluorescent signal were scored positive.

Under conditions wherein co-transfection of GFP-Ran with mCherry-α-tubulin or HA-GAPDH was performed, for quantitation of Ran transfer, we have chosen isolated fields, where a single cell was doubly transfected and the surrounding area did not have any doubly transfected cells. This was to avoid the possibility that the transfer would have occurred from another neighbouring Ran expressing cell.

For live cell counting, COS-7 cells were transfected with indicated constructs and presence of epifluorescence was monitored by direct visualization using an inverted microscope (Axiovert 200M; Carl Zeiss, Inc.) without fixing.

For quantitation, 15–30 random fields from three independent experiments were included. Data are expressed as mean ± SD. Statistical analysis has been performed using the Student’s t-test. *P*-values <0.05 were considered statistically significant.

## Results

**Fig 1:**
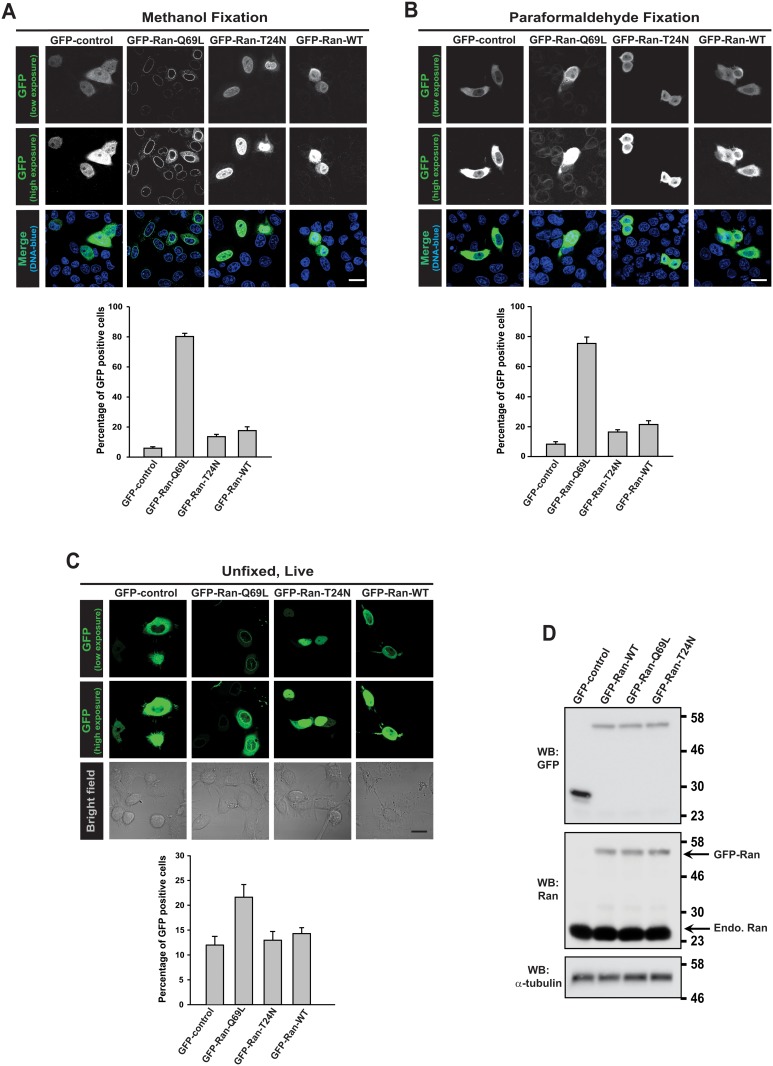
Ectopic expression of Ran GTPase. HeLa cells were transfected with indicated constructs for 24 h and fixed using methanol (A) or paraformaldehyde (B) and were processed for fluorescence microscopy. GFP is detected with a specific polyclonal antibody (green) and DNA was stained with Hoechst 33342 (blue). (C) COS-7 cells were transfected with indicated constructs for 24 h and the unfixed cells were directly visualized under fluorescence microscope. In all the above experiments, the adjacent respective graph represents quantitative data indicating the percentage of cells showing the GFP proteins and was derived from three independent experiments (in each experiment at least 100 cells were counted). Data are expressed as mean ± SD. Scale bar, 20 μm. (D) HeLa cells transfected with indicated constructs were lysed, separated on 10% SDS-PAGE and analysed by western blotting (WB) with GFP and Ran antibodies. α-tubulin was used as loading control. Molecular weights (in kDa) are shown in numbers.

HeLa cells were transfected with GFP, GFP-Ran-Q69L, GFP-Ran-T24N or GFP-Ran wild type (WT) constructs for 24 h and fixed with methanol and stained with GFP-specific antibodies. Intriguingly, we observed that transfection of HeLa cells with GFP-tagged version of RanQ69L (GTPase-deficient mutant, therefore predominantly GTP-bound) [40,41] resulted in its expression in almost all cells (Fig 1A). Ran-Q69L showed very distinct accumulation on NE as reported earlier for RanG19V, a similar mutant resistant to GTP hydrolysis [42]. On the contrary, under the same conditions, a lower number of cells expressed GFP control, GFP-Ran-WT or a Ran mutant deficient in nucleotide binding (Ran-T24N) [40,41,43]. The presence of Ran-Q69L in most cells would indicate many possibilities, one of them being transfer of the Ran protein from one cell to the other.

However, using methanol as fixative could lead to post fixation artefact as both fixation and permeabilization occur simultaneously. To rule out the possibility that presence of Ran-Q69L in many cells is not a post fixation artefact, we used paraformaldehyde (PFA), which fixes antigens by cross-linking and the cells were later permeabilized with triton X-100 for antibody penetration during immunostaining. Using this protocol also we observed that significantly higher number of cells was stained positive for GFP-Ran-Q69L compared to GFP-control, GFP-Ran-WT or GFP-Ran-T24N (Fig 1B). To completely rule out the possibility of any artefact during fixation, we transfected COS-7 cells with the constructs and the unfixed cells were directly visualized under fluorescent microscope and scored for the number of cells expressing GFP. As previously observed, significantly larger number of cells showed presence of GFP-Ran-Q69L as compared to other proteins (Fig 1C). The relative decrease in the percentage of transfected cells, when monitored by live counting (Fig 1C) as compared to when fixed with methanol (Fig 1A) or PFA (Fig 1B), would be due to difference in the sensitivity of detection. Live counting was based on detectable GFP epifluorescence, whereas, in methanol and PFA- fixed cells, GFP was detected using specific primary antibody and fluorescently labelled secondary antibodies (see Materials and Methods).

Transfected constructs expressed the proteins of expected molecular weight and their identities were confirmed by western analysis with GFP and Ran specific antibodies (Fig 1D). The relative intensities suggest that GFP-Ran mutants were expressed to much lesser extent as compared to endogenous Ran levels.

To rule out the possibility that the observed effect could be due to any artefact caused by lipo-based transfection methods, we alternatively expressed the constructs by transfecting HeLa cells with electroporation (nucleofection) and monitored the presence of GFP-tagged proteins (S1 Fig). The data suggested that transfection method did not make any difference to the pattern of Ran expression and / or distribution.

**Fig 2:**
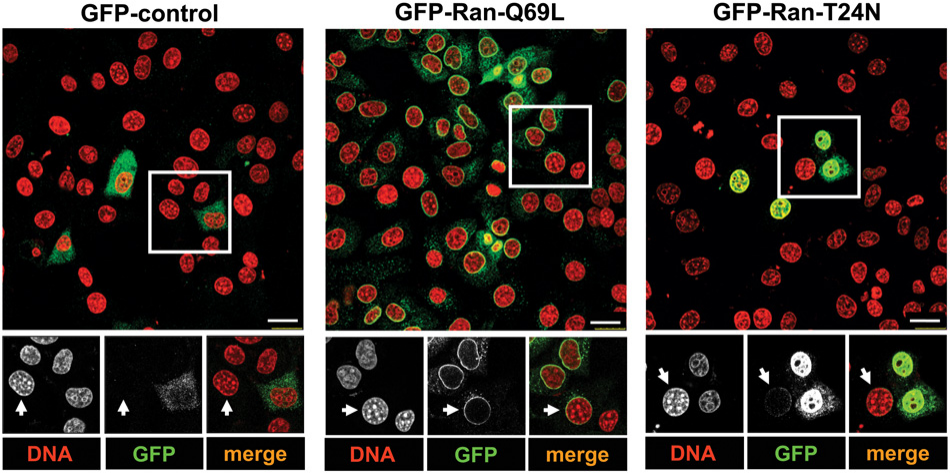
Inter-cellular transfer of Ran. HeLa cells were transfected with indicated constructs for 9 h and were then co-cultured with untransfected NIH3T3 cells for 18 h. Cells were stained with GFP antibody (green) and the DNA dye Hoechst 33342 (pseudocoloured in red). Arrows indicate NIH3T3 cells as detected by the characteristic punctate staining of the nucleus. Scale bar, 25 μm.

To test the possibility that Ran-Q69L transfer occurs between cells, transfected HeLa (human) cells were co-cultured with untransfected NIH3T3 (murine) cells, which could be distinguished from HeLa cells due to the punctate staining of the murine nucleus by Hoechst dye [44,45] (Fig 2, arrows). We could find that a significant number of co-cultured NIH3T3 cells displayed GFP-Ran-Q69L (~ 90%), as compared to GFP control (~2%), suggesting that the protein has been transferred from HeLa cells (donor) to NIH3T3 cells (recipient). Interestingly, GFP-Ran-T24N showed significantly less transfer (~16%) as compared to Ran-Q69L (Fig 2), indicating that the cell-to-cell distribution of Ran is GTP dependent.

**Fig 3:**
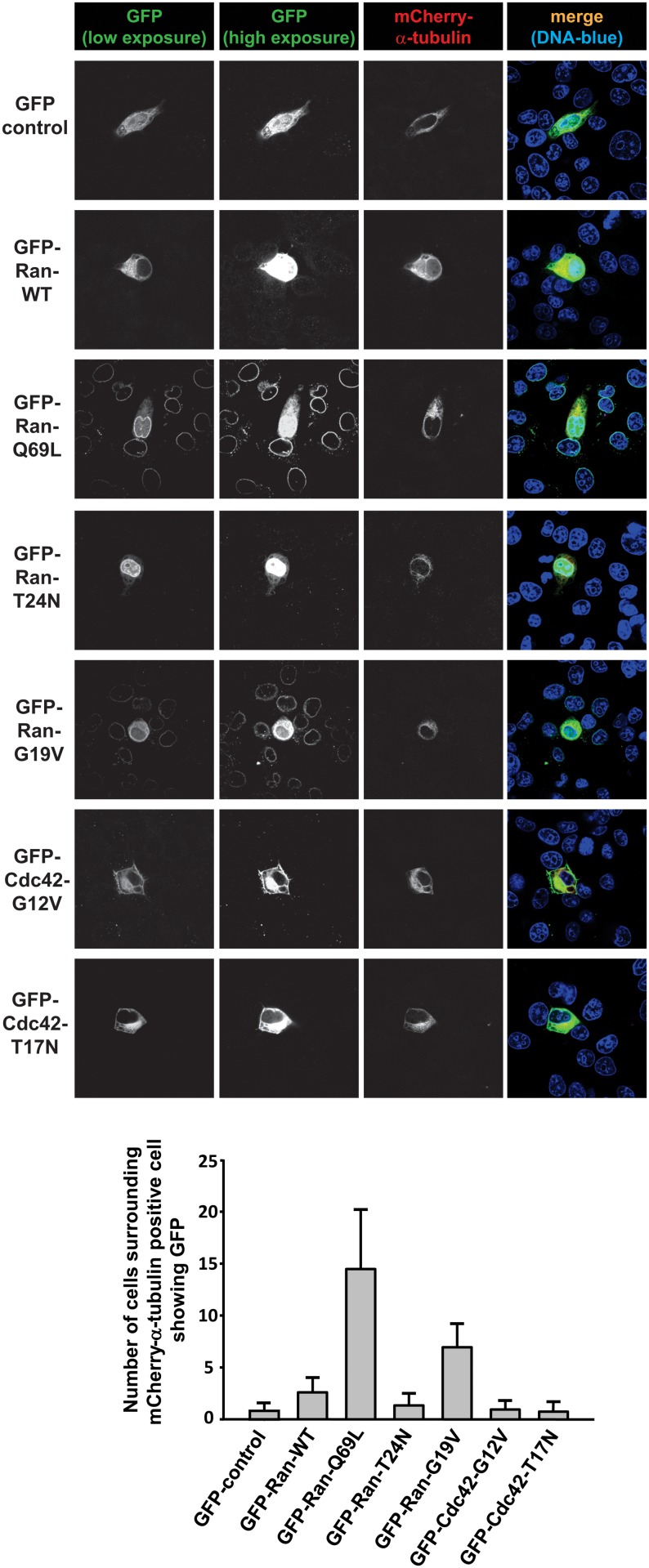
Transient transfection assay for inter-cellular transport of Ran. HeLa cells were co-transfected with mCherry-α-tubulin (transfection marker, red) and indicated GFP constructs (green) for 9 h. Cells were fixed and analysed for the presence of mCherry and GFP. DNA was stained in blue. Scale bar, 20 μm. Quantitative data showing the number of recipient cells displaying GFP staining surrounding the mCherry-α-tubulin positive donor cell. Cells were counted from 15 individual fields randomly across three independent experiments. Data are expressed as mean ± SD.

To further study the details of inter-cellular transport of Ran GTPase, we developed a simple assay by transiently co-transfecting GFP-Ran with mCherry-α-tubulin constructs for 9h, and by monitoring the presence of fluorescently tagged protein by microscopy. In GFP-control and mCherry-α-tubulin double transfected condition, we noticed that most of the transfected cells expressed both mCherry-α-tubulin and GFP (95.25%, 300 cells from three independent experiments) (Fig 3). However, when GFP-Ran-Q69L was co-expressed with mCherry-α-tubulin, we found many cells showing the presence of only GFP-Ran-Q69L. Moreover, we could identify the cell primarily transfected with Ran-Q69L (as judged by the presence of the transfection marker mCherry-α-tubulin, referred to as donor cell) displaying higher levels of GFP fluorescence (Fig 3). Strikingly, the recipient cells surrounding the mCherry-α-tubulin positive donor cell displayed a gradation of GFP fluorescence; the intensity being highest in the mCherry-α-tubulin positive donor cell and decreasing away from the donor cell as a function of distance. However, GFP-Ran-WT and GFP-Ran-T24N showed significantly less transfer as compared to Ran-Q69L (Fig 3). RanG19V, another mutant deficient in GTP hydrolysis and therefore GTP-bound, also showed enhanced transfer as compared to Ran-WT or Ran-T24N mutant. The reason for Ran-WT showing reduced transfer as compared to Ran-Q69L or Ran-G19V could be because Ran-WT exists mostly in GDP-bound form in the cytoplasm. The above results show that the GTP-bound Ran specifically gets distributed from cell to cell.

The specificity of Ran GTPase transfer was also confirmed by the fact that mutants of Cdc42, another Ras-like GTPase, showed no such inter-cellular distribution (Fig 3). We further quantitated the extent of transfer of wild type and different Ran mutants, by counting the number of cells showing GFP fluorescence surrounding the donor cell (Fig 3, lower panel). Within a period of 9–10 h post transfection, Ran-Q69L (GTP-Ran) showed maximum cell-to-cell transfer as compared to control GFP.

Alternatively, we used HA-Ran constructs to verify the inter-cellular transport of Ran, and to rule out if the earlier observation was due to any artefact owing to the use of a large tag such as GFP. The results suggested that HA-Ran-G19V showed significantly higher level of cell-to-cell transfer as compared to HA-Ran-T24N or HA-Ran-WT (S2 Fig). This confirmed that GTP-Ran displayed efficient cell-to-cell transfer irrespective of the tags used.

To ascertain the GTP-dependence, we made use of the temperature sensitive RCC1 (RanGEF) mutant cell line tsBN2 [5]. We tested the transfer of Ran under conditions of RCC1 loss (hence defect in GTP loading of Ran) upon shifting to non-permissive temperature (39.5°C). Within 3 h of shifting to non-permissive temperature, most of RCC1 was depleted in tsBN2 cells (S3 Fig). Interestingly, the transfer of Ran-Q69L was significantly reduced in tsBN2 cells under non-permissive temperature as compared to that in permissive temperature (S3 Fig). Collectively, these data demonstrate that Ran GTPase has the unique ability to move from cell to cell in a GTP-dependent manner.

**Fig 4:**
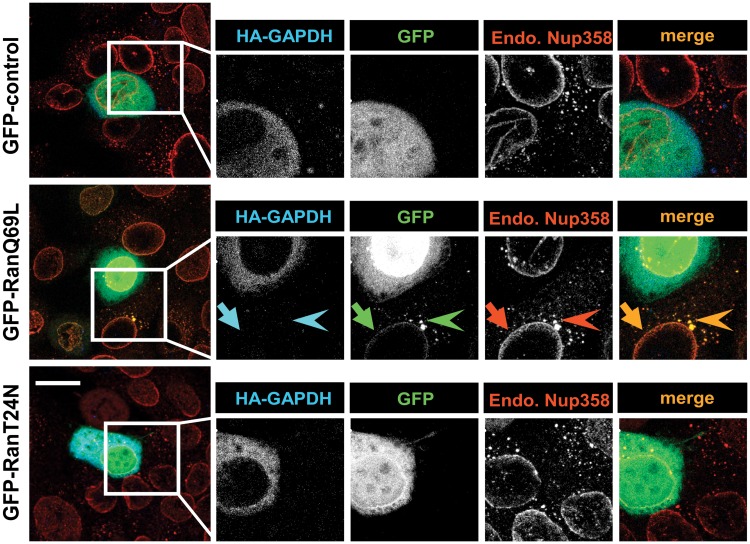
RanQ69L co-localizes with Nup358 in the recipient cells. HeLa cells were transfected with HA-GAPDH (transfection marker) and 2xGFP-control or Ran mutants as indicated. Cells were fixed and stained for HA-GAPDH (blue) and endogenous Nup358 (Endo.Nup358, red) using specific antibodies. GFP (green) was visualized by direct epifluorescence. Arrow indicates co-localization of RanQ69L with Nup358 on nuclear envelope and arrow head indicates co-localization in cytoplasmic punctae of recipient cells. Scale bar, 20 μm.

Nup358 has been identified as a major Ran-GTP binding protein in the cytoplasm [46,47]. A closer look at the distribution of Ran-Q69L revealed that it is predominantly present on the NE and in cytoplasmic punctae of the acceptor cell (Fig 4). Co-localization studies clearly indicated that Ran-Q69L is present along with Nup358 both on the NE and in the cytoplasmic punctae of the acceptor cells (Fig 4, arrow and arrowheads, respectively). GFP-control and GFP-Ran-T24N, however, did not show any significant co-localization with Nup358 in the cytoplasm. These data suggest that Ran-Q69L present in the recipient cell functionally retains the ability to bind to its partners such as Nup358.

**Fig 5:**
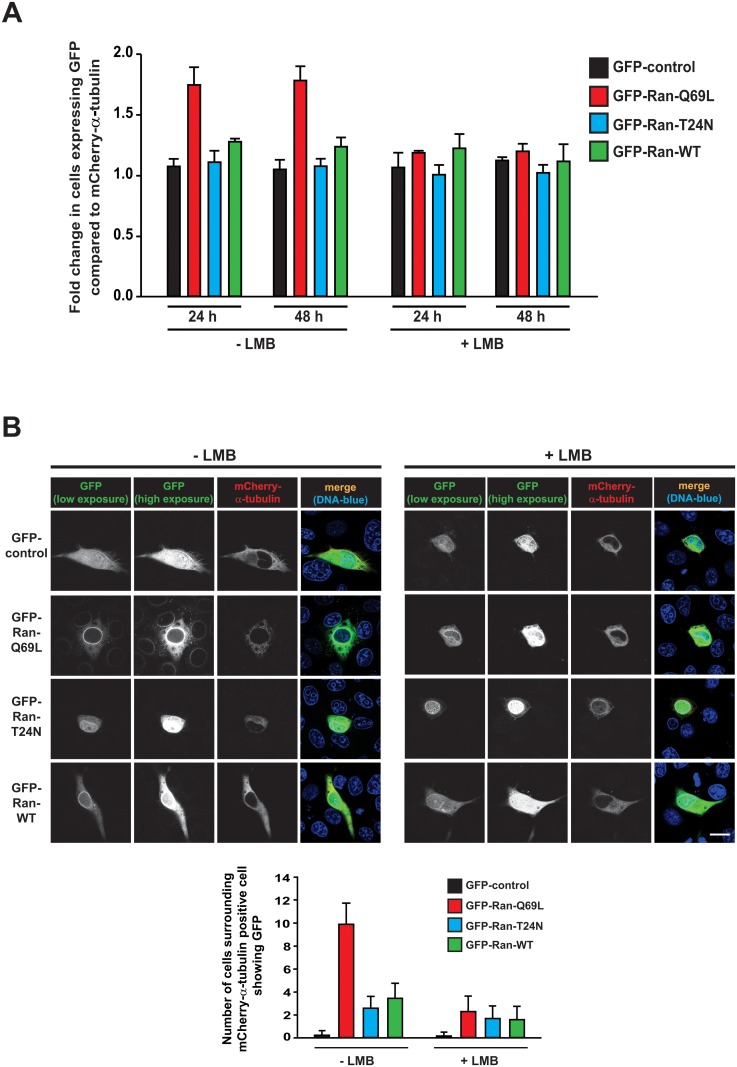
Leptomycin B treatment interferes with inter-cellular transport of Ran. (A) COS-7 cells were co-transfected with indicated GFP-constructs and mCherry-α-tubulin (transfection marker), and after 6h, were untreated (-) or treated (+) with leptomycin B (LMB, 5 ng/ml) and continued till the indicated time points (hours post-transfection). The inter-cellular transport of GFP-proteins was monitored by visualizing the unfixed cells directly under fluorescence microscope and calculating the fold increase in the number of GFP positive cells over the number of cells expressing the transfection marker mCherry-α-tubulin. Quantitative representation is shown. (B) LMB treatment in donor cells impairs inter-cellular transport of Ran. HeLa cells were co-transfected with HA-GAPDH and 2xGFP control or 2xGFP-Ran constructs as indicated. After 12 h, LMB treatment (5 ng/mL) was given for 12 h. Then the cells were washed, trypsinized and co-cultured with untreated HeLa cells (1:4 ratio of transfected to untransfected cells) and stained after 24 h for HA-GAPDH (transfection marker; blue) and endogenous Nup358 (red). GFP (green) was directly visualized by epifluorescence. Scale bar, 20 μm. *Lower Panel*: Quantitative representation of inter-cellular transport of Ran when untreated (-) and LMB treated (+) transfected (donor) cells were co-cultured with untransfected cells. The quantitation was performed as mentioned for Fig 3, except that cells were counted from 30 different fields across three independent experiments. Data are expressed as mean ± SD.

CRM1, an important RanGTP binding protein, is involved in the export of cargos from the nucleus to the cytoplasm. We were interested to test if leptomycin B (LMB), a known inhibitor of the export receptor CRM1 that affects its interaction with RanGTP [48], impaired cell-to-cell transfer of Ran. We co-transfected COS-7 cells with GFP-control, GFP-Ran-Q69L, GFP-Ran-T24N or GFP-Ran-WT and mCherry-α-tubulin (transfection marker) and monitored the cell-to-cell transfer using live microscopy at different time intervals in the absence or presence of LMB. We found that LMB significantly interfered with cell-to-cell transfer of Ran-Q69L (Fig 5A). Moreover, siRNA mediated depletion of CRM1 in HeLa cells significantly impaired the inter-cellular transfer of Ran (S4 Fig).

We wished to delineate if LMB treatment specifically affected donor cells from transferring Ran to the recipient cells. To address this, as LMB binds irreversibly to CRM1 [49], we treated cells expressing GFP-Ran proteins and mCherry-α-tubulin (transfection marker) with or without LMB and later co-cultured with untreated cells. The results suggested that when donor cells were treated with LMB, Ran-Q69L transfer to the recipient cells was specifically affected as compared to that in LMB-untreated cells (Fig 5B). This suggested that Ran transfer would depend on its interaction with CRM1.

## Discussion

This is the first report showing that any cellular GTPase possesses the ability to move from cell to cell. Our results demonstrate that Ran GTPase, a critical player in nucleo-cytoplasmic transport, is distributed from cell to cell in a GTP-dependent fashion. We also show that the inter-cellular transport requires interaction with CRM1. The important questions that need to be resolved include i) what is the molecular mechanism by which Ran gets distributed between cells and ii) what is the functional significance of such an inter-cellular transport. While the answers to these obvious questions require further investigation, some of the possibilities are discussed below.

The known methods of inter-cellular transfer of molecules/organelles involve microvesicles (exosomes and shedding vesicles) [27–29], TNTs [25,26] and non-conventional secretion and internalization [35,36]. It is possible that Ran GTPase might take any of the routes to travel between cells. Interestingly, protein profiling studies have suggested Ran to be present in the exosomes secreted by many cell types [50–54]. However, the possibility that Ran gets distributed through TNTs / microvesicles or involving non-conventional secretion similar to that reported for a class of proteins such as homeoproteins [35,36], needs to be confirmed by future work.

The most significant question is why does Ran get distributed between cells? The export complex, consisting of the cargo and export receptor (such as CRM1) in association with RanGTP, is believed to be released in the cytoplasm at the immediate vicinity of NPC through RanGAP1-mediated hydrolysis of GTP on Ran. As the GTP-Ran mutants (Q69L and G19V) showed dramatic ability to get distributed between cells, we speculate that some of the export complexes would escape GTP hydrolysis and could be transferred to the neighbouring cells. The export cargos could potentially be proteins or a subset of RNAs regulated by the Ran pathway including miRNAs. There could be mechanisms to specifically protect the complex from hydrolysis of Ran GTP in the donor cells, but allow its hydrolysis and specific release of the cargos in the recipient cells. This potentially allows one cell to regulate processes in the neighbouring cells through Ran-mediated transfer of macromolecules between cells. Consistent with this, one possible reason why LMB treatment of donor cells interfered with transfer of Ran from donor to acceptor cells (Fig 5B) could be due to the inability of Ran to make a functional export complex.

Recent findings show that proteins/ mRNAs/ miRNAs present in the microvesicles secreted by some cells could be transferred to other cells and direct processes in the recipient cells [29–31]. This could also apply to transport of macromolecules between cells through TNTs or non-conventional secretion and internalization. Given the ability of Ran to move from cell to cell, its involvement in dictating such cell-cell communication is a very intriguing possibility.

Overexpression of Ran GTPase has been strongly associated with various cancers [55–60]. As recent findings appreciate the role of inter-cellular distribution of macromolecules in cancer progression [28,33,50,61,62], one exciting possibility, although speculative, is that these scenarios would involve the above discussed novel roles for Ran. The inter-cellular transport of Ran could also regulate stem cell functions mediated by the niche cells and the immune cell communications. We propose that the findings reported here have the potential to unravel intricacies of inter-cellular communication in diverse cellular contexts, particularly those possibly mediated through inter-cellular transport of Ran GTPase.

## Supporting Information

S1 Fig Ectopic expression of Ran GTPase upon nucleofection. *Upper panel*, HeLa cells were transfected with indicated plasmids by nucleofection. Twenty four hours later, cells were fixed with methanol and stained for GFP using specific antibodies (green). DNA was visualized by Hoechst 33342 staining (blue). Scale bar, 20 μm. *Lower panel*, Quantitative data showing the number of cells displaying GFP staining. Cells were counted from 30 individual fields randomly across three independent experiments. Data are expressed as mean ± SD.
(PDF)

S2 Fig Distribution of ectopically expressed HA-tagged version of Ran GTPase. *Upper panel*, HeLa cells were co-transfected with HA-Ran- G19V, HA-Ran-T24N or HA-Ran-WT and mCherry-α-tubulin as transfection marker. Nine hours later cells were fixed with methanol and stained for HA using specific antibodies (green). mCherry-α-tubulin (red) was detected by epifluorescence. DNA was visualized by Hoechst 33342 staining (blue). Scale bar, 20 μm. *Lower panel*, Quantitative data showing the number of recipient cells displaying GFP staining surrounding the mCherry-α-tubulin positive donor cell. Cells were counted from 30 individual fields randomly across three independent experiments. Data are expressed as mean ± SD.
(PDF)

S3 Fig Effect of RCC1 depletion on Ran transfer. (A) tsBN2 cells were grown at permissive temperature (32.5°C) and were transfected with indicated plasmids for 3 h. Cells were continued in permissive temperature or shifted to non-permissive temperature (39.5°C). Eight hours later cells were fixed with methanol and stained for GFP using specific antibodies (green). mCherry-α-tubulin (red) was detected by epifluorescence. DNA was visualized by Hoechst 33342 staining (blue). (B) tsBN2 cells were grown at permissive temperature or non-permissive temperature for 3 h and the level of RCC1 was monitored by western blotting. α-tubulin was used as loading control. (C) Quantitative data showing fold change in cells expressing GFP over mCherry-α-tubulin. Cells were counted from 30 individual fields randomly across three independent experiments. Data are expressed as mean ± SD.
(PDF)

S4 Fig Effect of CRM1 depletion on Ran transfer. (A) HeLa cells were transfected with control (siControl) or CRM1 specific (siCRM1) siRNA for 60 h. The cell lysates were analyzed for the level of CRM1 by western blotting. α-tubulin was used as loading control. (B) HeLa cells were transfected with control or CRM1-specific siRNA for 36 h and then co-transfected with indicated GFP and mCherry-α-tubulin constructs. Twenty four hours later, cells were fixed with methanol and stained for GFP using specific antibodies. mCherry-α-tubulin was detected by epifluorescence. Fold change in cells expressing GFP over mCherry-α-tubulin was determined. Cells were counted from 30 individual fields randomly across three independent experiments. Data are expressed as mean ± SD.
(PDF)
