# Supplementary material for: Inter-Cellular Transport of Ran GTPase
Source: PLoS One. 2015 Apr 20;10(4):e0125506. doi: 10.1371/journal.pone.0125506 (PMC4403925; doi:10.1371/journal.pone.0125506)
Supplement: S1 Fig — Upper panel, HeLa cells were transfected with indicated plasmids by nucleofection. Twenty four hours later, cells were fixed with methanol and stained for GFP using specific antibodies (green). DNA was visualized by Hoechst 33342 staining (blue). Scale bar, 20 μm. Lower panel, Quantitative data showing the number of cells displaying GFP staining. Cells were counted from 30 individual fields randomly across three independent experiments. Data are expressed as mean ± SD. (PDF) [file pone.0125506.s001.pdf]

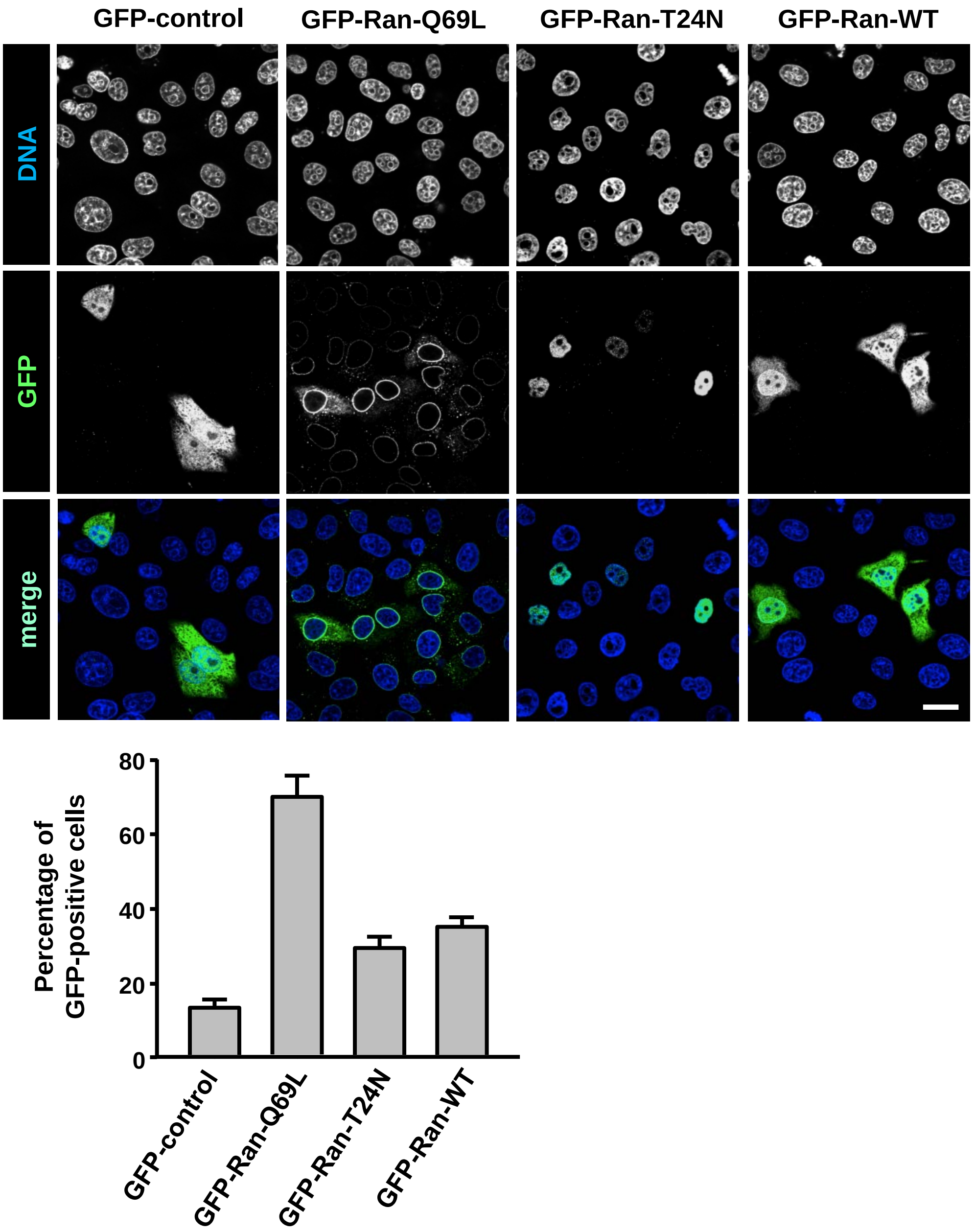

S1 Fig. Ectopic expression of Ran GTPase upon nucleofection. *Upper panel,* HeLa cells were transfected with indicated plasmids by nucleofection. Twenty four hours later, cells were fixed with methanol and stained for GFP using specific antibodies (green). DNA was visualized by Hoechst 33342 staining (blue). Scale bar, 20 μm. *Lower panel*, Quantitative data showing the number of cells displaying GFP staining. Cells were counted from 30 individual fields randomly across three independent experiments. Data are expressed as mean ± SD.
