# Supplementary material for: Inter-Cellular Transport of Ran GTPase
Source: PLoS One. 2015 Apr 20;10(4):e0125506. doi: 10.1371/journal.pone.0125506 (PMC4403925; doi:10.1371/journal.pone.0125506)
Supplement: S2 Fig — Upper panel, HeLa cells were co-transfected with HA-Ran- G19V, HA-Ran-T24N or HA-Ran-WT and mCherry-α-tubulin as transfection marker. Nine hours later cells were fixed with methanol and stained for HA using specific antibodies (green). mCherry-α-tubulin (red) was detected by epifluorescence. DNA was visualized by Hoechst 33342 staining (blue). Scale bar, 20 μm. Lower panel, Quantitative data showing the number of recipient cells displaying GFP staining surrounding the mCherry-α-tubulin positive donor cell. Cells were counted from 30 individual fields randomly across three independent experiments. Data are expressed as mean ± SD. (PDF) [file pone.0125506.s002.pdf]

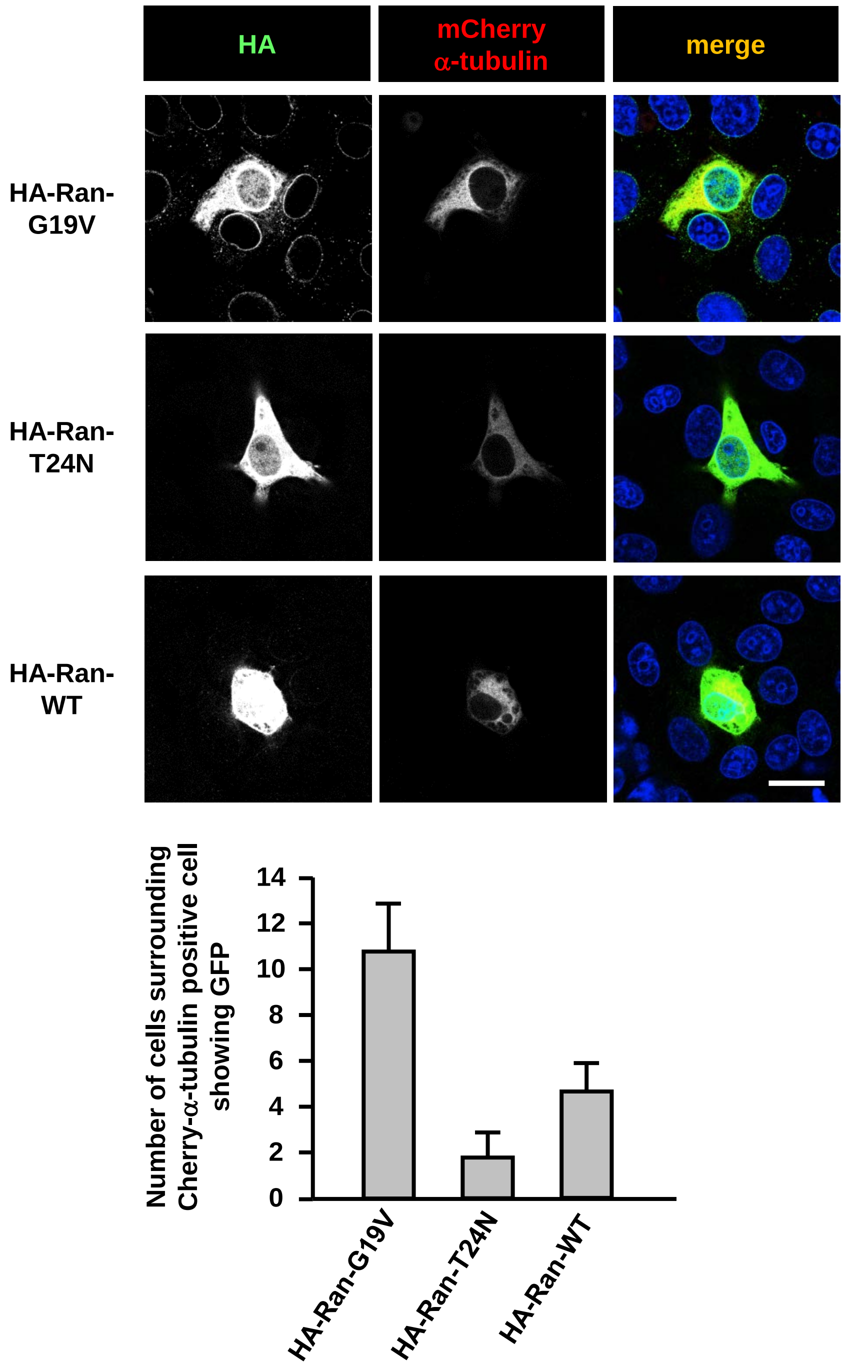

S2 Fig. Distribution of ectopically expressed HA-tagged version of Ran GTPase. *Upper panel,* HeLa cells were co-transfected with HA-Ran- G19V, HA-Ran-T24N or HA-Ran-WT and mCherry-α-tubulin as transfection marker. Nine hours later cells were fixed with methanol and stained for HA using specific antibodies (green). mCherry-α-tubulin (red) was detected by epifluorescence. DNA was visualized by Hoechst 33342 staining (blue). Scale bar, 20 μm. *Lower panel*, Quantitative data showing the number of recipient cells displaying GFP staining surrounding the mCherry-α-tubulin positive donor cell. Cells were counted from 30 individual fields randomly across three independent experiments. Data are expressed as mean ± SD.
