# Supplementary material for: Inter-Cellular Transport of Ran GTPase
Source: PLoS One. 2015 Apr 20;10(4):e0125506. doi: 10.1371/journal.pone.0125506 (PMC4403925; doi:10.1371/journal.pone.0125506)
Supplement: S3 Fig — (A) tsBN2 cells were grown at permissive temperature (32.5°C) and were transfected with indicated plasmids for 3 h. Cells were continued in permissive temperature or shifted to non-permissive temperature (39.5°C). Eight hours later cells were fixed with methanol and stained for GFP using specific antibodies (green). mCherry-α-tubulin (red) was detected by epifluorescence. DNA was visualized by Hoechst 33342 staining (blue). (B) tsBN2 cells were grown at permissive temperature or non-permissive temperature for 3 h and the level of RCC1 was monitored by western blotting. α-tubulin was used as loading control. (C) Quantitative data showing fold change in cells expressing GFP over mCherry-α-tubulin. Cells were counted from 30 individual fields randomly across three independent experiments. Data are expressed as mean ± SD. (PDF) [file pone.0125506.s003.pdf]

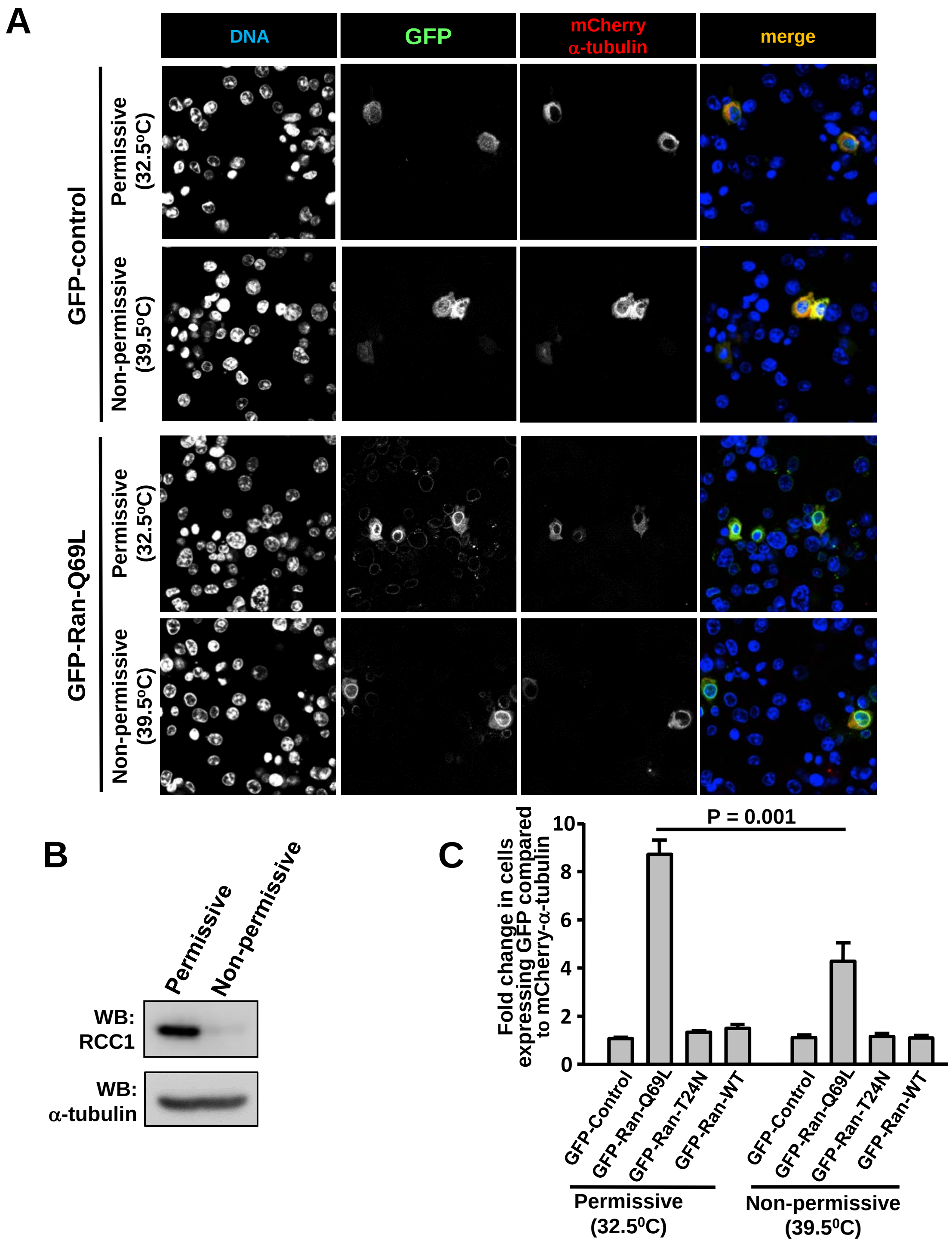

S3 Fig. Effect of RCC1 depletion on Ran transfer. (A) tsBN2 cells were grown at permissive temperature (32.5^0C) and were transfected with indicated plasmids for 3 h. Cells were continued in permissive temperature or shifted to non-permissive temperature (39.5^0C). Eight hours later cells were fixed with methanol and stained for GFP using specific antibodies (green). mCherry-α-tubulin (red) was detected by epifluorescence. DNA was visualized by Hoechst 33342 staining (blue). (B) tsBN2 cells were grown at permissive temperature or non-permissive temperature for 3 h and the level of RCC1 was monitored by western blotting. α-tubulin was used as loading control. (C) Quantitative data showing fold change in cells expressing GFP over mCherry-α-tubulin. Cells were counted from 30 individual fields randomly across three independent experiments. Data are expressed as mean ± SD.
