# Supplementary material for: Inter-Cellular Transport of Ran GTPase
Source: PLoS One. 2015 Apr 20;10(4):e0125506. doi: 10.1371/journal.pone.0125506 (PMC4403925; doi:10.1371/journal.pone.0125506)
Supplement: S4 Fig — (A) HeLa cells were transfected with control (siControl) or CRM1 specific (siCRM1) siRNA for 60 h. The cell lysates were analyzed for the level of CRM1 by western blotting. α-tubulin was used as loading control. (B) HeLa cells were transfected with control or CRM1-specific siRNA for 36 h and then co-transfected with indicated GFP and mCherry-α-tubulin constructs. Twenty four hours later, cells were fixed with methanol and stained for GFP using specific antibodies. mCherry-α-tubulin was detected by epifluorescence. Fold change in cells expressing GFP over mCherry-α-tubulin was determined. Cells were counted from 30 individual fields randomly across three independent experiments. Data are expressed as mean ± SD. (PDF) [file pone.0125506.s004.pdf]

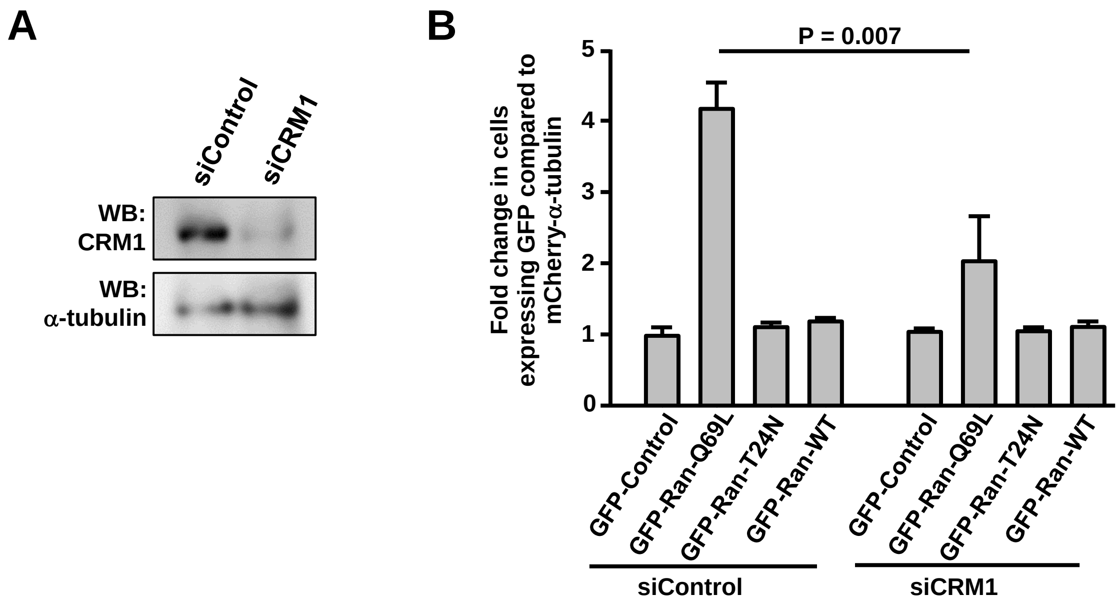

S4 Fig. Effect of CRM1 depletion on Ran transfer. (A) HeLa cells were transfected with control (siControl) or CRM1 specific (siCRM1) siRNA for 60 h. The cell lysates were analyzed for the level of CRM1 by western blotting. α-tubulin was used as loading control. (B) HeLa cells were transfected with control or CRM1-specific siRNA for 36 h and then co-transfected with indicated GFP and mCherry-α-tubulin constructs. Twenty four hours later, cells were fixed with methanol and stained for GFP using specific antibodies. mCherry-α-tubulin was detected by epifluorescence. Fold change in cells expressing GFP over mCherry-α-tubulin was determined. Cells were counted from 30 individual fields randomly across three independent experiments. Data are expressed as mean ± SD.
